# Laryngeal vs. Esophageal Acid-Mediated Injury in Bulimia Nervosa: A Systematic Review

**DOI:** 10.7759/cureus.116303

**Published:** 2026-09-15

**Authors:** Sheilan Pouri, Sophia Cresitello, Kristina Shaw, Alina Zgardau, Ahmad Ahmadi

**Affiliations:** 1 Medicine, Dr. Kiran C. Patel College of Osteopathic Medicine, Nova Southeastern University, Davie, USA; 2 Medicine, Schulich School of Medicine & Dentistry, Western University, London, CAN; 3 Anatomy/Neuroanatomy/Dentistry, Dr. Kiran C. Patel College of Osteopathic Medicine, Nova Southeastern University, Fort Lauderdale, USA

**Keywords:** acid-mediated injury, bulimia nervosa, dysphonia, esophageal mucosa, functional anatomy of the larynx, gastroesophageal reflux disease (gerd), laryngopharyngeal reflux (lpr), multidisciplinary care team, pepsin enzyme, vocal fold

## Abstract

Bulimia nervosa (BN) is a psychiatric eating disorder that can potentially be life-threatening and is characterized by recurrent binging and/or purging. During purging episodes that involve self-induced vomiting, laryngopharyngeal reflux (LPR) can occur as gastric acid is forcefully pushed through the esophageal sphincter and anteriorly to the acid-sensitive laryngopharyngeal tissue. While the esophageal mucosal lining can tolerate repeated exposure to acid before sustaining significant erosion, the laryngeal mucosa exhibits greater sensitivity, reacting to even mild exposure with inflammation. LPR has been shown to be associated with hoarseness, capillary dilation, enlargement of soft tissue, arytenoid, and thyroid cartilages, and thick mucus over the larynx. Individual studies have also been conducted on how the larynx sustains injury at higher pH levels compared to the esophagus. However, no literature review has yet synthesized why exactly the larynx experiences disproportionate vulnerability due to anatomical, histological, and acid-tolerance mechanisms. As such, this review aims to consolidate evidence on how injury to the larynx is greater than that to the esophagus among BN patients engaging in self-induced vomiting. The 2020 Preferred Reporting Items for Systematic Reviews and Meta-Analyses (PRISMA) guidelines were used for this analysis. A systematic review was conducted using PubMed, Web of Science, and Embase between 1957 and 2026 using two separate Boolean search strings: one primary search for primary, clinical, and population-level evidence on BN and/or laryngopharyngeal/esophageal pathology, and a secondary search to collect mechanistic and background literature. Eligible studies examined acid-mediated injury, laryngeal pathology, esophageal pathology, or comparative mucosal damage in BN or LPR. Excluded studies included non-peer-reviewed sources and opinion pieces unrelated to BN, LPR, or acid-mediated mucosal injury. Through the primary Boolean search (Search 1), nine studies met eligibility criteria and were used as primary evidence. These nine studies were supplemented by 12 mechanistic sources that were found through the second Boolean search string (Search 2); 10 foundational sources were identified through reference list chaining and included in this review. A total of 31 sources were included in the synthesis of this review, with two additional references used purely for methodological purposes (the PRISMA 2020 statement and the Newcastle-Ottawa Scale), for a total of 33 cited references. Across primary studies, 51 unique subjects were represented, 30 of whom had a formal BN diagnosis. An additional 380 subjects were represented throughout the mechanistic/supporting review literature examining general LPR and/or GERD populations. Across studies, laryngeal mucosa sustained earlier and more severe pathological changes compared to esophageal mucosa among BN patients. These findings indicate the larynx is disproportionately injured in BN due to markedly lower acid-exposure tolerance thresholds, thinner epithelium, and reduced buffering capacity of the mucosa. Prospective studies comparing laryngeal and esophageal mucosal outcomes among BN patients are warranted. Laryngeal symptoms in clinical settings, such as hoarseness, may help improve early diagnostic precision and implement interventional strategies, supporting otolaryngologic involvement in multidisciplinary care for this understudied patient population.

## Introduction and background

Bulimia nervosa (BN) is an eating disorder characterized by recurrent episodes of compensatory behaviors, including purging (self-induced vomiting), laxative use, and excessive exercise [1-3]. This condition has one of the highest rates of mortality of any psychiatric disorder, with a standardized mortality ratio of 1.9 (95% CI: 1.4-2.6; a value above 1 indicates excessive mortality). As a result of the low pH of stomach acid and the repetitive action of vomiting multiple times a day, these factors have been linked to pathological disruptions in throat anatomy and are linked to BN [4].

The larynx is a funnel-shaped, cartilaginous organ positioned anteriorly at C3-C6 vertebrae and directly superior to the esophagus. It consists of three major protective structures that redirect ingested material heading towards the airway: the vocal cords, the epiglottis, and the laryngeal ventricles. During episodes of self-induced vomiting, elevated intra-abdominal pressure and reflex relaxation of the upper and lower esophageal sphincters facilitate the retrograde propulsion of gastric contents through the esophagus [5,6]. This reversal of normal peristaltic flow allows acid to enter the hypopharynx, where the aerodigestive tract diverges into the anterior larynx and posterior esophagus. Direct contact is made with the laryngeal inlet, including the epiglottis and vocal folds [7]. Under normal conditions, the upper esophageal sphincter functions as a barrier, preventing refluxate from entering the pharynx. This protective mechanism is overcome during vomiting, increasing the risk of laryngeal exposure to gastric acid [5,6]. Disruption of these mucosal protective mechanisms can cause anatomical changes and thus can lead to various downstream laryngeal pathologies [8].

Esophageal and laryngeal mucosa have sharply contrasting differences in their thresholds for acid exposure. The esophageal mucosa has a superior capacity for handling frequent exposure due to structural adaptation, thick stratified squamous epithelium, and great bicarbonate buffering capacity. Experimental literature has shown that even at a low pH of 1.3, acid alone does not cause esophagitis in the absence of pepsin, and this is due to pepsin being denatured at a pH >7.0. This indicates that both low pH and active pepsin are required to cause esophageal injury [9]. By contrast, the laryngeal mucosa lacks these defense mechanisms, causing vulnerability to the same acidic concentrations as the esophagus [10]. The laryngeal epithelium has been shown to be substantially more acid-sensitive than the esophagus, with as little as three exposures to gastric contents a week having the potential to cause injury to the laryngeal mucosa [11]. The larynx is also regionally heterogeneous in its lining: the posterior commissure and true vocal folds are protected by non-keratinized stratified squamous epithelium, whereas all other regions of the larynx are covered by ciliated pseudostratified columnar epithelium, which lacks the same level of barrier integrity. This leaves certain areas of the larynx more vulnerable to acid-mediated injury [12]. Laryngopharyngeal reflux (LPR) has been associated with hoarseness, capillary dilation, enlargement of soft tissue, arytenoid, and thyroid cartilages, and thick mucus over the larynx [10,13].

Despite a substantial amount of literature examining esophageal reflux and other complications of BN patients, literature regarding BN-related laryngeal reflux is comparatively limited. While there are individual studies on laryngeal mucosa damage due to acid-mediated exposure, recent systematic reviews have synthesized modifications of human vocal-fold tissue in LPR with findings including inflammation, intercellular-space dilation, pepsin-induced DNA damage, and downregulation of carbonic anhydrase III [14]. That said, no review has directly compared laryngeal and esophageal susceptibility to acid-exposure thresholds specifically in this vulnerable patient population [15,16]. It remains unclear how the specific mechanisms, including anatomical, histological, and pH-tolerance mechanisms outlined in previous literature, collectively cause injury to the larynx, disproportionately, and whether these characteristics contribute to increased laryngeal vulnerability. Thus, the goal of this review is to synthesize evidence in primary and review literature on how the larynx disproportionality sustains greater injury than the esophagus in patients with BN, with particular emphasis on lower acid tolerance thresholds and reduced mucosal protective defenses. This review also aims to improve the early diagnosis and management of BN-related laryngeal injury by highlighting laryngeal injury as a potential early clinical manifestation and providing a framework for future studies to help guide multidisciplinary care teams with diagnosis and treatment protocols [17].

## Review

Methods

Study Design and Search Strategy

This systematic review was conducted in accordance with the 2020 Preferred Reporting Items for Systematic Reviews and Meta-Analyses (PRISMA) guidelines [18]. The 2020 PRISMA flow diagram template from the official PRISMA statement website was used to report the selection of studies. Two comprehensive searches for literature were conducted across three electronic databases: PubMed, Web of Science, and Embase from 1957 to 2026. The 1957 start date was chosen to align with the Bernstein and Baker acid profusion test, which established esophageal acid sensitivity and remains a foundational study for LPR, throat anatomy, and throat pathology in BN patients. No literature predating this study is expected to provide data relevant to this review. The search was last conducted in June 2026.

Primary search terms included combinations of keywords including “bulimia nervosa”, “purging”, “self-induced vomiting”, “vocal fold”, “larynx”, “pepsin”, “reflux”, “esophagus”, “dysphonia”, “hoarseness”, “gastroesophageal reflux”, “GERD”, “LPR”, and “laryngopharyngeal reflux”. The first and primary Boolean search string applied across all three databases was used to target clinical and population-level evidence on BN and laryngopharyngeal and esophageal pathology as follows: ("bulimia nervosa" OR "bulimia" OR "self-induced vomiting" OR “purging”) AND (“larynx” OR "vocal fold" OR “hoarseness” OR “dysphonia” OR “dysphagia” OR "laryngopharyngeal reflux" OR “LPR” OR “pepsin” OR "GERD" OR “reflux” OR “esophagus” OR “Barrett”). This search returned 169 records in PubMed, 445 records in Embase, and 50 records in Web of Science (total n = 664), as detailed in Figure 1. The second Boolean search string was conducted to obtain foundational and anatomical literature that contributes to the mechanistic rationale of this systematic review’s hypothesis. This search was conducted only using the title field, and term pairings were used to maintain specificity: ("laryngopharyngeal" AND "reflux") OR ("laryngeal" AND "pepsin") OR ("esophageal" AND "mucosa") OR ("vocal fold" AND "reflux") OR ("larynx" AND ("mucosa" OR "immunology")) OR ("esophagus" AND "pepsin"). Field syntax was adopted per each database's requirements (PubMed: “[ti]”; Embase: “:ti”; Web of Science: field search “title” was selected). PubMed had 1,800 records, Embase had 2,262, and Web of Science had 3,117 (total n = 7,179). Given the dense results of this search, it was not screened in full, and supporting literature was instead identified through a targeted review of highly relevant titles and abstracts to support the anatomical and biological findings of this review. To reiterate, this search was intended only to identify background literature rather than primary studies for systematic inclusion. Of the 12 mechanistic sources cited in this systematic review, 10 were retrieved across all three databases. Two were results found only in PubMed (Koufman; Shilpa et al.). Ten additional sources not found by either Boolean search string, comprising foundational GI and BN medical complications, anatomical background, foundational diagnostic methodology references, and histological literature, were identified through manual screening of articles’ reference lists.

**Figure 1:**
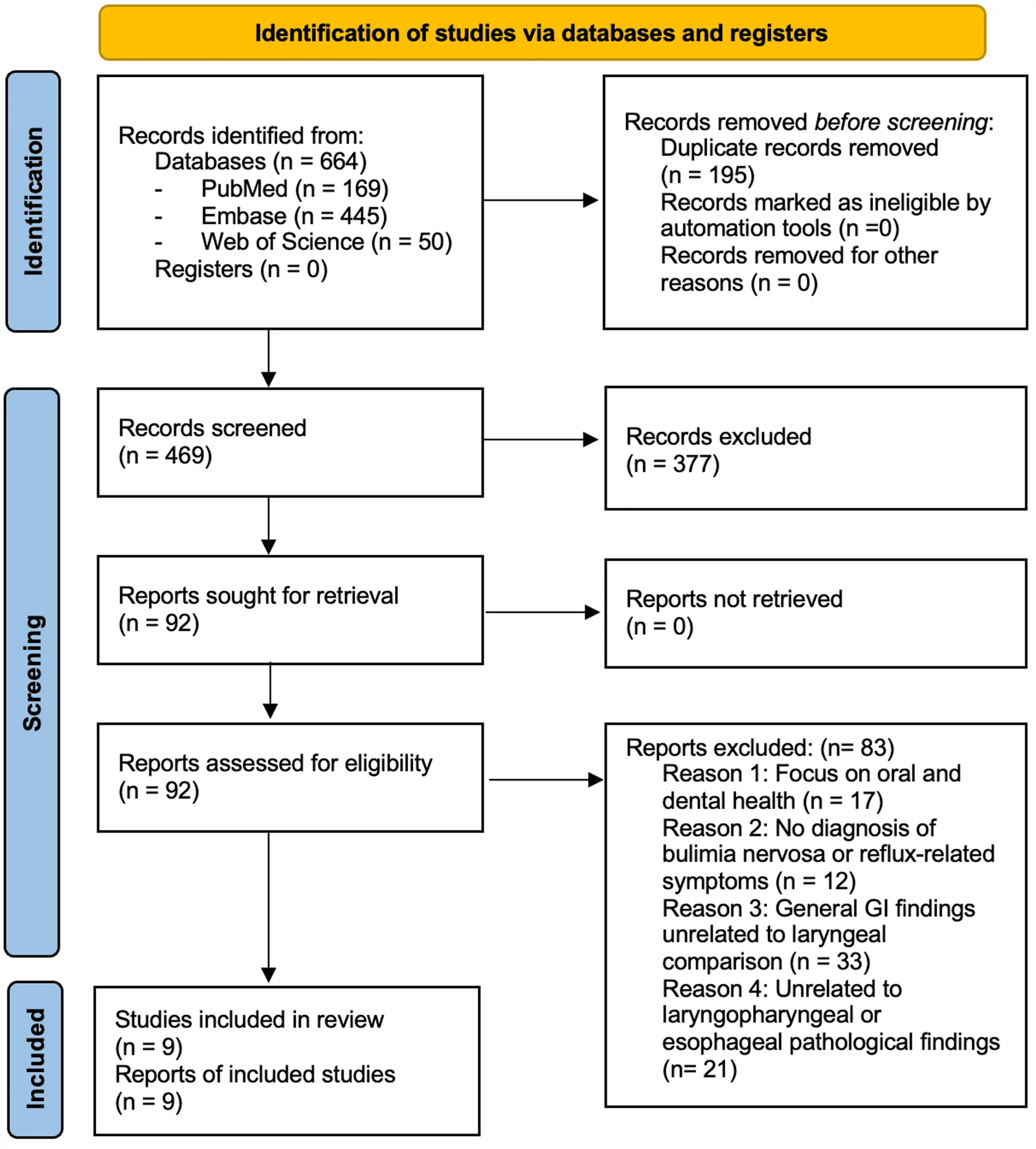
PRISMA 2020 study selection flow diagram that includes study identification, screening, and inclusion process for this systematic review's primary Boolean search string (Search 1) Records from Search 1 were identified across three electronic databases: PubMed, Embase, and Web of Science. Records were screened according to the eligibility and inclusion process outlined in this flowchart and the Methods section. This flow diagram is a depiction of the primary Boolean search (Search 1) only. A second Boolean search string (Search 2) was used to retrieve 12 foundational anatomical and mechanistic literature reviews; reference-list chaining identified 10 additional sources. These are reported separately in the Methods and are not included in this flowchart because they did not undergo formal eligibility screening. This decision is consistent with manual screening and Search 2’s roles as supplements to Search 1’s study selection. Adapted from: [18]. BN: bulimia nervosa; NOS: Newcastle-Ottawa Scale; PRISMA: Preferred Reporting Items for Systematic Reviews and Meta-Analyses.

To reiterate, sources were grouped into three tiers: (1) primary evidence, consisting of nine studies, was systematically selected from Boolean Search 1 and underwent full eligibility screening and quality assessment; (2) secondary and supplemental sources, consisting of 12 mechanistic studies identified through Boolean Search 2 and 10 studies identified through manual reference-list screening, which provided background information including anatomical, physiological, and histological context and did not undergo formal scoring or full eligibility screening that tier 1 was subject to; and (3) methodological sources, consisting of the PRISMA 2020 statement and the Newcastle-Ottawa Scale (NOS). Tier one studies contributed to the formal systematic synthesis of evidence, while tiers two and three only provided important background and contextual information and should therefore not be considered primary evidence.

Eligibility Criteria

Inclusion criteria of study selection required that studies be (1) peer-reviewed published articles in the English language that examined (2) acid-mediated injury, laryngeal pathology, esophageal pathology, or comparative mucosal damage in BN or LPR; and (3) original research study data, systematic reviews or clinical reviews that presented data critical to the research question, and narrative reviews for background and contextual information (not used as primary sources of evidence). The narrative reviews and mechanistic sources were identified through the second Boolean search string and manual screening of references, which is why they were not subject to the same eligibility criteria as the first Boolean search string. Data extraction was conducted independently by three reviewers who evaluated titles, abstracts, and full texts.

Excluded studies included those that were non-peer-reviewed sources, opinion pieces, or studies unrelated to BN, LPR, laryngeal pathology, esophageal pathology, acid-reflux mechanisms of purging, or acid-mediated laryngeal and esophageal pathology.

Data Extraction

Outcomes and study characteristics were extracted into a standardized Google Sheet template. Discrepancies in screening or data extraction were resolved by a fourth reviewer or via discussion and consensus among the three reviewers. A systematic review was performed with a narrative synthesis. Collected data were synthesized to assess patterns of laryngeal injury in the context of acid exposure and to evaluate proposed mechanisms for greater laryngeal susceptibility relative to the esophagus, including pH tolerance thresholds, histological markers and endoscopic findings. Outcomes assessed qualitative and quantitative evidence of anatomical and histological disruptions in esophageal and laryngeal tissue following acid exposure. Due to the substantial heterogeneity of included study designs, populations, exposure models, and outcome measures, quantitative pooling was not feasible. Therefore, a synthesis including P-values and quantitative outcomes was performed, given the findings from original studies.

Literature Selection Process

Records from the first Boolean search string (n=664) were imported into Rayyan (Rayyan Systems Inc., Cambridge, MA, USA) for the removal of duplicate literature and general screening. A total of 195 duplicated records were removed prior to the screening process, yielding 469 unique records. Screening was performed by four independent reviewers. Of these 469 records, 377 were excluded, leaving 92 records for the full-text screening process. All 92 records were successfully found and assessed for eligibility. Ultimately 83 studies were excluded due to (1) focus on oral and dental health (n = 17); (2) no diagnosis of BN or reflux-related symptoms (n = 12); (3) general GI findings unrelated to laryngeal comparison (n = 33); and (4) unrelated to laryngopharyngeal or esophageal pathological findings (n = 21). These nine studies comprise the selected primary evidence included in this systematic review from Boolean Search 1 and are the only sources that underwent full eligibility screening and formal risk-of-bias assessment; this complete selection process is outlined in Figure 1. As described previously, these nine sources were supplemented by 12 mechanistic sources found with Boolean Search 2: 10 foundational studies selected via reference-list chaining and two methodological references.

Quality and Bias Assessment

Quality and risk-of-bias assessment were independently evaluated by four reviewers using the NOS for human observational studies identified through search string 1, which included the following information: Author(s), year of publication, selection methodology, study model, sample size and exposure groups, population, key finding(s), if any, and indication of laryngopharyngeal and/or esophageal damage relevant to the question this review aims to answer [19]. Included observational studies were scored using the NOS selection, comparability, and outcomes with a maximum score of nine (selection 4, comparability 2, outcome/exposure 3). Studies scoring ≥7 were considered high-quality, ≥4 were considered moderate quality, and anything below a four was considered low quality and excluded.

The key quantitative findings from each study were recorded to directly support the research question of laryngeal versus esophageal acid tolerance. Laboratory-based experiments were not subjected to the NOS and were instead assessed qualitatively for methodological selection, statistical reporting, experimental model, source of tissue, reproducibility, and transparency of reporting. Key quantitative findings were extracted from each study, including, but not limited to, mucosal pH thresholds, percentage of epithelial tissue damage, and frequency of reflux episodes that cause mucosal injury. Systematic, narrative, and experimental/clinical reviews were not subjected to formal scoring, as these pieces of literature were mainly utilized as a source of contextual information. The NOS was used specifically to identify risk-of-bias assessment among human observational studies rather than as a screening tool. Following this method, the Search 2 mechanistic sources and manually screened reference-list sources did not undergo formal eligibility or risk-of-bias assessment. Discrepancies were resolved by consensus or through consultation with a fifth reviewer.

Results

Characteristics of Selected Studies

The 31 included sources (excluding two methodological sources) comprised of comprehensive reviews (n=18), case reports or case series (n=2), observational cohort studies (n=5), and experimental studies (n=5), with one additional mixed-methods report (Table 1). Across primary studies focusing on BN patients, 51 subjects were clearly represented, 30 of whom had a formal diagnosis of BN (Sunnetci: 10 + Ferreira: 11 + Rothstein: 8 + Gouda: 1). An additional 380 subjects are also represented in the supporting foundational literature (Koufman: 225 GERD patients, Shilpa: 100 LPR/GERD patients, Bernstein & Baker: 55 esophagitis patients). The remaining participants were either controls, had an LPR/GERD diagnosis without BN, or, in one study (Maciejewska et al.), had a diagnosis of anorexia nervosa and were included in this review solely as contextual evidence rather than primary evidence [20]. The complete selection process per PRISMA guidelines is outlined in Figure 1.

**Table 1:** Characteristics of all 33 studies included in this systematic review examining laryngeal versus esophageal mucosal injury in patients with bulimia nervosa The table reports the first author, year of publication, search source, evidence category, study model, sample size, population, key findings, and indication of laryngopharyngeal and/or esophageal pathology that is relevant to this systematic review’s research question. Each source is characterized by whether it was identified through the primary systematic search (Boolean search string 1), secondary search for mechanistic literature (Boolean search string 2), or manual reference-list screening, as detailed in the Methods section. Only the studies identified from Search 1 are listed as primary evidence and underwent formal risk-of-bias assessment. Full citation details are provided in the reference list. BN: bulimia nervosa; CAIII: carbonic anhydrase isoenzyme III; DIS: dilated intercellular spaces; ENT: ear, nose, and throat; LPR: laryngopharyngeal reflux; GERD: gastroesophageal reflux disease; GI: gastrointestinal; GRBASI: grade, roughness, breathiness, asthenia, strain, instability (dysphonia scale); LES: lower esophageal sphincter; LPR: laryngopharyngeal reflux; NOS: Newcastle-Ottawa Scale; PPI: proton pump inhibitor; PRISMA: Preferred Reporting Items for Systematic Reviews and Meta-Analyses; UES: upper esophageal sphincter; VHI-10: Voice Handicap Index-10.

First Author + Year of Publication	Search	Evidence Category	Study Model	Sample Size	Population	Key Findings	Indication of Laryngopharyngeal and/or Esophageal Damage
van Eeden AE et al., 2021 [1]	Manual	Background/Mechanistic	Narrative review	Not applicable	Adolescent and young adult males and females	BN has seen an overall decline in incidence. BN has a lifetime prevalence rate of 3% and carries an increased mortality risk compared to the general population.	Focuses on the rising pediatric prevalence of BN rather than explicitly quantified indication of laryngopharyngeal and/or esophageal damage.
Nitsch A et al., 2021 [2]	Search 2	Background/Mechanistic	Comprehensive clinical review article	Not applicable	Wide demographic of patients diagnosed with BN and participating in binging/purging activities.	Patients who present with frequent purging behaviors are at increased risk for dental erosion, systemic medical complications and severe life-threatening fluid and electrolyte imbalances.	Patients who partake in consistent self-induced vomiting causes direct damage and harm to oral and pharyngeal mucosa, which manifests as cough, chronic pharyngitis, hoarseness, and dysphagia.
van Hoeken D et al., 2020 [3]	Manual	Background/Mechanistic	Literature review	Not applicable	Wide global demographics focusing on young females, adolescents and psychiatric inpatients.	Eating disorders result in significantly reduced quality of life, higher health costs, elevated risk of obstetric complications and over 3 million deaths globally per year, with a marked 10% increase in patients with BN.	Indication of laryngopharyngeal and/or esophageal damage not explicitly quantified. The article focuses on the macroscopic scope of systemic disabilities experienced by patients with BN.
Sunnetci G et al., 2024 [4]	Search 1	Primary evidence	Case-control study	20 participants; 10 patients diagnosed with BN and 10 age-matched healthy controls without eating disorders	Young adults with similar age profile, half as a control group and half diagnosed with BN	Patients with BN showed elevated acoustic disturbance values (jitter and shimmer) and significantly higher subjective Voice Handicap Index-10 (VHI-10) scores compared to healthy controls (P<0.05), showing clear voice impairment and subjective vocal discontent.	Repetitive episodes of self-induced vomiting lead to a localized laryngopharyngeal reflux (LPR) mechanism that directly aggravates the patient’s vocal cords. Patients with BN showed a significantly higher Reflux Symptom Index and abnormal vocal cord examinations.
Desai J et al., 2023 [5]	Manual	Background/Mechanistic	Comprehensive clinical textbook/education review	Not applicable	Broad demographic highlighting etiologies that include reflux-induced injuries and malignant strictures in adults and accidental corrosive ingestion in pediatric cohorts	Peptic strictures caused chronic gastroesophageal reflux disease (GERD) lead to high risk clinical complications such as direct esophageal perforation and aspiration-induced asthma.	Pathological formation of structures indicates structural esophageal damage resulting in fibrosis, chronic inflammation, neoplasia or fibrosis that directly exposes the mucosal and submucosal layers of the esophagus resulting in permanent loss of tissue elasticity.
Lang V et al., 1997 [6]	Manual	Background/Mechanistic	Physiological and anatomical review article	Not applicable	General mammalian and human physiology models	The Upper Esophageal Sphincter (UES) is a complex and highly compliant musculature that works to maintain resting tone via different protective glossopharyngo-vagal and vago-vagal reflexes.	Focuses on typical physiological barriers and the role the UES serves to prevent damaging esophagopharyngeal reflex and airway aspiration.
Bruss DM et al., 2023 [7]	Manual	Background/Mechanistic	Comprehensive anatomical and clinical textbook review	Not applicable	General human anatomy and physiological models	The laryngopharynx is an essential structural division that bifurcates anteriorly into the larynx and posteriorly into the esophagus. Highlighting the 3 distinct tissue layers that comprises the pharyngeal wall and their role in protection and function.	The laryngopharynx is a highly sensitive site for tissue damage resulting from LPR and the damage to the thin mucosal layer results in chronic inflammatory remodeling, squamous cell carcinomas and localized direct mucosal irritation.
Denholm M et al., 2011 [8]	Search 2	Background/Mechanistic	Literature review	Not applicable	Individuals diagnosed with eating disorders – primarily BN	Chronic self-induced episodes of vomiting lead to elevated threshold for lower esophageal sphincter relaxation, damage to upper gastrointestinal motility, and dental erosion.	Repeated exposure to gastric acid and mechanical stress secondary to purging leads to esophageal mucosal deterioration, esophagitis and retrograde acidic flow causing laryngopharyngeal damage and irritation.
Hirschowitz, B.I., 1999 [9]	Search 2	Background/Mechanistic	Synthesis of experimental animal studies + author’s past clinical studies	Many studies included: the largest clinical cohorts cited include 51 stricture patients vs. 50 controls, and 26 Barrett's esophagus patients vs. 27 controls	General GERD and esophagitis patients; experimental animal models (rabbit, cat, dog esophagus)	Esophagitis and GERD require pepsin to be present alongside acid, as acid alone does not damage esophageal tissue even at pH 1.3. Pepsin remains stable and reactivatable up to pH 6.9, denaturing only above pH 7. Gastric acid must be above a pH of 4 for at least 75% of the day to promote tissue healing.	Specific pH/pepsin threshold for esophageal injury established. The experimental resistance profile can be used to directly compare Bulmer et al.’s pH/pepsin thresholds.
Forney KJ et al., 2016 [10]	Search 2	Background/Mechanistic	Comprehensive clinical review article	Not applicable	All demographics displaying purging behaviors including self-induced vomiting, laxative abuse and diuretic abuse	All forms of purging can cause life-threatening electrolyte and fluid abnormalities but self-induced vomiting is responsible for the most severe systemic medical conditions.	Repetitive episodes of self-induced vomiting exposes the upper gastrointestinal and respiratory tracts to unhealthy levels of gastric acid which leads to tissue fibrosis, chronic inflammation, mechanical trauma, pre-cancerous cellular modifications and LPR symptoms such as cough, dysphagia, and hoarseness
Sirin et al., 2019 [11]	Search 2	Background/Mechanistic	Editorial review article	Not applicable	General patients with suspected laryngopharyngeal reflux (LPR) and GERD	LPR and GERD were identified as distinct pathological reflux disorders with different mechanisms and tissue susceptibilities. The authors emphasized that the laryngeal epithelium is substantially more acid-sensitive than esophageal tissue, with as few as three acid exposures per week capable of causing laryngeal injury.	LPR causes mucosal edema, hyperemia, chronic dysphonia, throat clearing, cough, and inflammatory remodeling of the vocal folds due to acid and pepsin exposure. The article further highlights that laryngeal tissue sustains injury at higher pH levels and with shorter acid exposure durations than esophageal tissue.
Squier CA et al., 2001 [12]	Search 2	Background/Mechanistic	Biological/anatomical review article	Not applicable	Human oral and esophageal mucosal physiology models	Oral and esophageal mucosa function as protective epithelial barriers against mechanical and chemical injury. Nonkeratinized esophageal tissue demonstrates greater permeability and susceptibility to injury compared to keratinized mucosa.	Chronic acid exposure compromises epithelial integrity, reduces healing capacity, promotes inflammation, fibrosis, ulceration, and increases mucosal vulnerability to reflux-mediated injury and infection.
Cui N et al., 2024 [13]	Search 2	Background/Mechanistic	Comprehensive clinical review article	Not applicable	Broad population of individuals who display episodes of self-induced vomiting	Episodes of self-induced vomiting led to LES dysfunction and UES failure resulting in both esophageal and laryngopharyngeal damage.	The laryngopharyngeal anatomy is more susceptible to damage and injury compared to the esophagus, secondary to the slower healing rate of its tissue and thinner layer of mucosa.
Chen G & Lechien JR, 2025 [14]	Search 2	Background/Mechanistic	Literature review	Not applicable	Adults with LPR undergoing vocal-fold tissue analysis	LPR vocal-fold tissue analysis consistently shows cell inflammation, cell-junction (E-cadherin) proteolysis, dilated spaces intercellularly, pepsin-associated DNA damage, downregulation of carbonic anhydrase III and protective mucins (e.g., MUC2, MUC4, MUC5AC).	Direct documentation of laryngeal mucosal injury. Reflux exposure erodes epithelial barrier and buffer enzymes in human tissues, corroborating histological basis for laryngeal mucosal vulnerability.
Ferreira CP et al., 2010 [15]	Search 1	Primary evidence	Case-control study	22 women (11 with purging bulimia nervosa, 11 healthy controls)	Women aged 18–37 years; patients with purging-type bulimia nervosa and healthy controls	Patients with bulimia demonstrated significantly higher rates of laryngeal abnormalities, increased GRBASI dysphonia scores, and elevated acoustic disturbance measures compared with controls. Thick laryngeal secretions, interarytenoid mucosal thickening, and glottic abnormalities were common findings.	Repeated self-induced vomiting exposes laryngeal tissues to gastric acid and pepsin, resulting in chronic mucosal irritation, inflammation, edema, interarytenoid hypertrophy, vocal fold lesions, and dysphonia. Frequent vomiting also contributes to lower esophageal sphincter relaxation and esophagitis, facilitating laryngopharyngeal reflux-related injury.
Balata P et al., 2008 [16]	Search 1	Primary evidence	Literature review	Not applicable	Adult and adolescent females with BN	23 reviewed papers; 3 of which specifically identified BN as a factor for voice disorders in adult women. No link found for voice changes in adolescent female BN patients.	Recurrent vomiting exposes both the larynx and oral cavity to gastric contents by a mechanisms that is analogous to GERD and can cause disruptions in voice-quality and laryngeal mucosa.
Sansone et al., 2012 [17]	Manual	Background/Mechanistic	Review article	Not applicable	Young females with self-induced vomiting eating disorders	Hoarseness may serve as a clinical indicator of reflux that is caused by self-induced vomiting, pointing to bulimia.	Possible laryngopharyngeal reflux and voice/laryngeal irritation from acid exposure.
Page MJ et al., 2021 [18]	Methodological	Methodological	Consensus-based reporting guideline and methodological statement	Not applicable	Not applicable	PRISMA 2020 statement provides a checklist and revised flow diagrams created to show advances in methods for appraising, selecting, identifying, and synthesizing studies.	Not applicable
Lo C.K. et al., 2014 [19]	Methodological	Methodological	Methological source	182 cohort studies eligible	Cohort studies from 2008-2011	This provides a quality-assessment tool for evaluating risk of bias and scoring for non-randomized studies with cohort designs and case-control designs.	Not applicable
Maciejewska et al., 2016 [20]	Search 1	Primary evidence	Preliminary prospective observational study	41 adolescent girls	Adolescent girls with anorexia nervosa	Voice changes including breathiness, shorter phonation time, abnormal vocal acoustics, altered vocal jitter and frequency.	Laryngeal structure and vocal apparatus go through functional and structural changes due to malnutrition and hormonal effects.
Bulmer DM, et al., 2010 [21]	Search 1	Primary evidence	Prospective translational study	Ex vivo porcine laryngeal tissue was tested. 5 anatomical sites tested (ventricles, vocal folds, posterior commissure, supraglottic, and subglottic mucosa).	Porcine laryngeal mucosa	Tissue in an Ussing chamber model was exposed to pH of 2 and 4 with and without pepsin for one hour. All tissues resisted damage at pH of 4 (without pepsin) except the subglottic mucosa. Only the posterior commissure resisted pH of 2 (with pepsin). Damage was found to require the presence of pepsin, as acid at a pH of 4 alone did not cause injury.	Established that laryngeal mucosa injury in LPR is due to the presence of pepsin and that the degree of injury correlated with anatomical site. The subglottic mucosa was the most vulnerable to injury while the posterior commissure was the most resistant.
Erickson E. et al., 2010 [22]	Search 2	Background/Mechanistic	Ex vivo experimental study	52 porcine vocal folds	Healthy porcine laryngeal/vocal fold tissue models exposed to simulated reflux conditions	Exposure to acidic pepsin and acid-only reflux conditions caused rapid and irreversible reductions in vocal fold transepithelial resistance (up to 77% decline within 30 minutes), indicating increased epithelial permeability and barrier dysfunction.	Simulated reflux directly compromised vocal fold epithelial integrity and increased epithelial “leakiness,” supporting the concept that gastric acid and pepsin exposure cause laryngeal mucosal injury, inflammatory remodeling, and heightened susceptibility to further reflux-related tissue damage.
Koufman J., 1991 [23]	Search 1	Primary evidence	Combined human cohort + experimental animal study	225 otolaryngology patients; canine model	Otolaryngology patients with suspected GERD that were evaluated for reflux	24 hour double-probe pH monitoring established occult GERD is a significant reason that the laryngopharyngeal tissue develops inflammatory and neoplastic diseases. ; the experimental canine model findings demonstrated that combined acid and pepsin exposure increases likelihood of producing laryngeal injury.	The mechanism of how acid and pepsin injure the laryngeal mucosa, especially in LPR, was established.
Rothstein S et al., 1998 [24]	Search 1	Primary evidence	Small cohort/case-series study	8 participants	Adult professional signers diagnosed with BN who have secondary vocal changes and dysphonia	LPR was objectively shown in 100% of the study’s singers with BN displaying direct association of bulimia induced vomiting with vocal performance disorders.	Chronic episodes of self-induced vomiting exposes the laryngeal tissue to the low pH of gastric acid resulting in structural damage leading to anatomical modifications that cause vocal fold edema, tissue hypertrophy, hoarseness and polypoid changes.
Matsuo K et al., 2008 [25]	Manual	Background/Mechanistic	Narrative review	Not applicable	Human swallowing physiology and dysphagia models	Normal swallowing requires coordinated oral, pharyngeal, laryngeal, UES, and esophageal function to safely transport a bolus while protecting the airway. Structural or functional abnormalities can impair swallowing efficiency and airway protection.	Dysfunction of the larynx, pharynx, UES, or esophagus may result in dysphagia, aspiration, bolus retention, regurgitation, and reflux-associated injury. Esophageal strictures related to GERD, inflammation-induced UES dysfunction, and regurgitation of acidic contents can contribute to laryngopharyngeal irritation and tissue damage.
Bernstein & Baker, 1958 [26]	Manual	Background/Mechanistic	Clinical diagnostic test	55 patients (67 total tests performed)	Veterans Administration Hospital patients being evaluated for esophagitis vs angina pectoris grouped into: endoscopic evidence of esophagitis - (n=10), pseudoesophagitis (positive history of esophagitis but no endoscopic evidence; n=12), normal esophagus (no history of esophagitis or endoscopic evidence; n=21), and not endoscoped (n=12)	Clinical symptoms of esophagitis reproduced vis the acid profusion test in 22 of 26 patients with a history and/or endoscopic findings. No findings of esophagitis in 28 out of 29 patients who denied history of esophagitis. The acid profusion test accurately distinguished esophageal from cardiac pain.	Established the esophageal mucosa as an acid-sensitive structure and experimental exposure was a valid and reproducible diagnostic marker of esophagitis.
Thibeault S et al., 2009 [27]	Search 2	Background/Mechanistic	Mucosal immunology review article	Not applicable	Human and mammalian laryngeal mucosal models	The larynx serves as an immunologic crossroads between the gastrointestinal and respiratory tracts and is highly exposed to inhaled and refluxed irritants. Chronic laryngeal inflammation alters epithelial immune architecture and mucosal defense mechanisms.	Laryngeal mucosa is highly vulnerable to chronic inflammatory remodeling from repeated exposure to refluxate and irritants, contributing to chronic laryngitis, mucosal irritation, and impaired vocal function.
Gill et al., 2005 [28]	Manual	Background/Mechanistic	Cross-sectional Western blot study	54 laryngeal biopsy specimens from 18 patients with LPR	Patients diagnosed with LPR	E-cadherin, pepsin, and CAIII in laryngeal biopsies were analyzed. CAIII was absent in 64% of LPR patients.	Identified a molecular defense of CAIII that is absent in LPR and causes pepsin to reduce the integrity of the laryngeal epithelial barrier.
Johnston N et al., 2007 [29]	Search 2	Background/Mechanistic	In-vitro laboratory study	Laboratory assays of purified human pepsin under different pH conditions	In-vitro human pepsin enzyme	At pH 7.4, human pepsin is stable and can be reactivated by acid exposure; indicating pepsin is not destroyed at pH’s near neutral once it has been introduced to the tissue.	Pepsin still retains injury-causing potential even in weak acidic or neutral pH environments, unlike acid alone which requires a low pH to cause damage to mucosa.
Orlando RC., 2010 [30]	Search 2	Background/Mechanistic	Physiological and pathophysiological review article	Not applicable	General human and animal esophageal physiology models	Gastric acid and pepsin disrupt the esophageal epithelial barrier by increasing paracellular permeability, producing dilated intercellular spaces (DIS), inflammation, cellular necrosis, and impaired tissue repair mechanisms associated with GERD progression.	Acid reflux directly damages the esophageal mucosa through epithelial barrier breakdown, inflammation, cellular injury, and chronic remodeling, contributing to erosive esophagitis, Barrett’s esophagus, and persistent reflux-related tissue injury.
Shilpa C et al., 2019 [31]	Search 1	Primary evidence	Clinical cohort study	100 patients in ENT outpatient clinic	Patients in ENT outpatient department with GERD symptoms classified by outcome into: LPR only (n=23), GERD only (n=19), combined LPR and GERD (n=40), and normal/control (n=18)	The most common symptoms of LPR patients included hoarseness, globus sensation, and heartburn. Patients with positive findings were treated with PPIs and followed for 3 months	LPR and GERD frequently co-occur but are clinically distinguishable. While laryngeal and esophageal reflux manifestations are relatable, they are not interchangeable diagnostic entities
Mendell D., et al., 2001 [32]	Manual	Background/Mechanistic	Literature review	Not applicable	Patients with bulimia nervosa engaging in recurrent self-induced vomiting	Repeated vomiting behaviors in BN were associated with dental erosion, altered gag reflexes, sensory abnormalities, delayed swallow initiation, abnormal bolus transport, and oropharyngeal swallowing dysfunction.	Chronic regurgitation of gastric acid and repeated gag stimulation contribute to oral, pharyngeal, and laryngeal mucosal irritation, tissue injury, ulceration, granuloma formation, sensory receptor dysfunction, and potential swallowing impairment secondary to repeated acid exposure and mechanical trauma.
Gouda et al., 2022 [33]	Search 1	Primary evidence	Case report	1 participant	47-year-old male patient with long-standing BN and self-induced vomiting	A patient with a 17-year history of binge eating and self-induced vomiting presented with severe GERD grade D and biopsy-confirmed Barrett’s esophagus with intestinal metaplasia.	Chronic reflux and repetitive acid exposure from prolonged purging behavior resulted in severe esophageal mucosal injury, Barrett’s metaplasia, and increased risk for esophageal adenocarcinoma.

All selected studies examined the comparative influence of BN on laryngeal versus esophageal tissue, though varied in scopes, interventions, and outcomes, as detailed in Table 1. Not all studies provided a direct comparison between laryngeal and esophageal tissues, but critical evidence was contributed to one or more components of the comparison outlined in this review’s research question. Studies ranged from specific clinical investigations of adult professional singers and pediatric populations diagnosed with BN to broader systemic and narrative reviews of the medical complications associated with self-induced vomiting and LPR. Some studies focused exclusively on the histological architecture and immunology markers of laryngeal mucosa, while others examined the broader physiologic effects of purging across multiple organ systems. Clinical studies utilized high-resolution interventional tools, such as laryngeal videostroboscopy and 24-hour dual-probe pH-metry, to assess in vivo laryngeal acid exposure, while experimental models assessed ex vivo tissues and their response to both acid and pepsin exposure. This includes tracking transepithelial resistance in porcine vocal fold tissues and Ussing chamber apparatuses (measures nutrient and drug transport across epithelial tissues to assess barrier functions and membrane properties) in porcine laryngeal mucosa [21,22]. Study outcomes consistently reported structural and functional damage to laryngeal and esophageal mucosa. Notably, laryngeal mucosa sustained injury after only four reflux episodes daily, while the esophageal mucosa can endure up to 50 refluxate episodes without epithelial damage [13,23]. These outcomes also included objective laryngeal findings such as subepithelial hemorrhage and posterior commissure hypertrophy, as well as the degradation of biochemical markers such as laryngeal carbonic anhydrase III [4,13,15,24,25]. While self-induced vomiting produces esophageal outcomes such as tears or esophagitis, the esophagus can withstand more frequent and prolonged acidic insults than the larynx before experiencing any significant structural damage [2,8,21,26].

Comparative Mechanisms

Anatomical differences: Anatomical distinctions have been shown to predispose the larynx to greater acid-mediated injury. Bulmer et al. characterized the mucosal lining of the larynx as thinner than the esophagus (in supporting mechanistic literature), rendering it more susceptible to low gastric pH [21]. The laryngeal aditus opens into the hypopharynx before joining the esophagus, positioning it in the retrograde path of gastric expulsion. Under normal conditions, the larynx should remain segregated from gastric acidity and relies on active epiglottis deflection, vocal cord closure, and elevation of the voice box to protect it (Figure 2) [7,25]. Matsuo and Palmer observed that the larynx aditus opening is in the direct path of receiving retrograde gastric contents, particularly after forceful vomiting, which can overwhelm the anatomical defenses in place [25].

**Figure 2:**
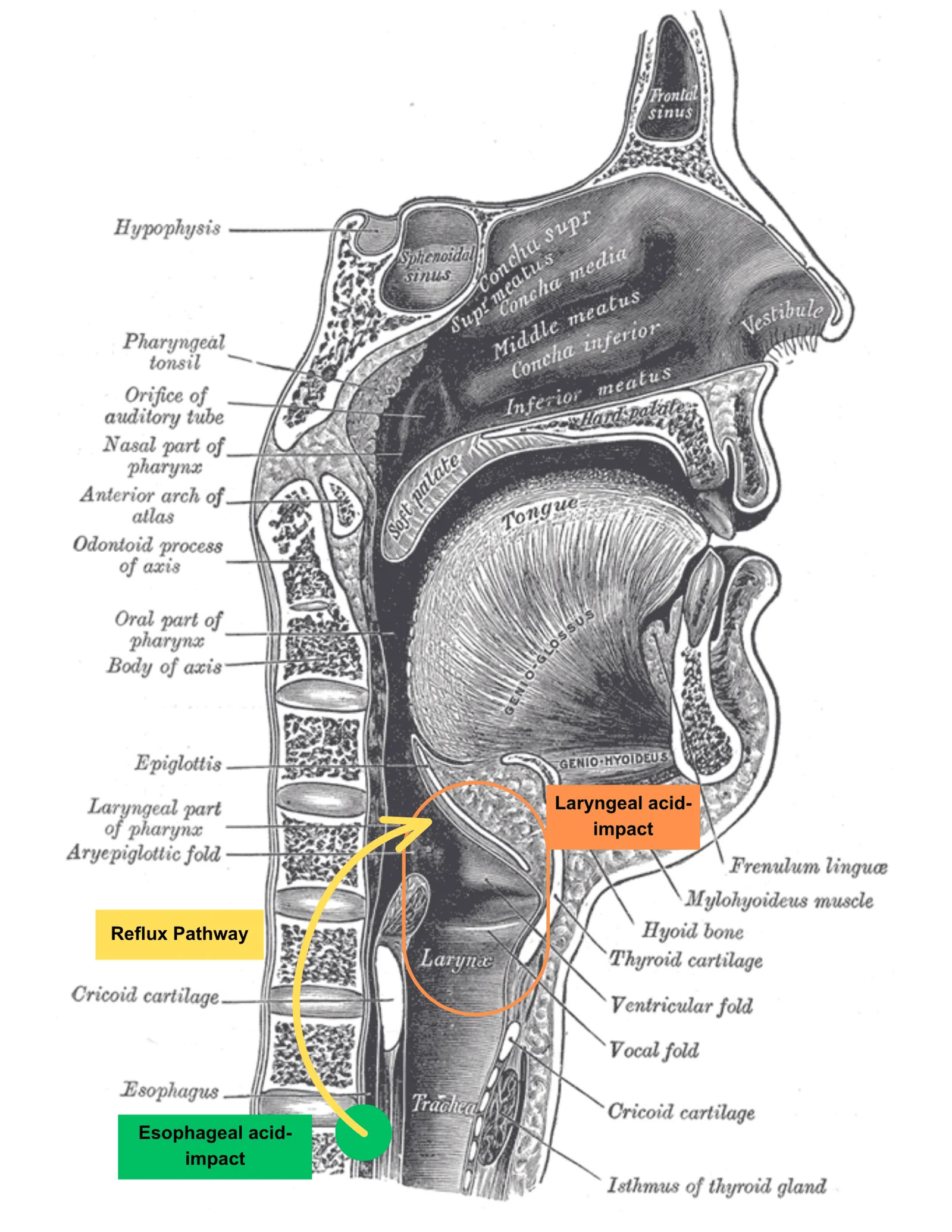
Acid refluxate pathway of the larynx versus esophagus in bulimia nervosa* During episodes of purging, patients with BN forcefully expel gastric contents containing acid and pepsin retrogradely through the esophagus, causing direct contact with the hypopharynx. Acidic contents can then spill into the laryngeal inlet if the airway-protective reflexes are overwhelmed. The reflux pathway (yellow), as well as the sites of esophageal (green) and laryngeal (orange), are annotated as such in the figure. *Base anatomical illustration obtained from Henry Vandyke Carter, public domain, via Wikimedia Commons (originally published in Gray's Anatomy). Annotations are added by authors to illustrate the refluxate pathway described in this review. BN: bulimia nervosa

In supporting background literature, Squier and Kremer identified that the esophagus is composed of thick stratified squamous epithelium cells that undergo rapid turnover, perform high rates of bicarbonate buffering, and maintain continuous epithelial barrier integrity, all of which are features that collectively aid in acid-exposure resistance [7,12,25]. In the larynx, variation in epithelium type may explain site-specific injury patterns seen in BN patients. Non-keratinized stratified squamous epithelium sites such as the posterior commissure and true vocal folds are comparatively resistant, whereas the epiglottis, laryngeal ventricles, and vestibular folds are more prone to injury [12,14]. Collectively, these structural findings establish that the larynx is anatomically predisposed to acid-mediated injury, particularly during BN-related purging episodes. The larynx lacks the anatomical defenses that protect the esophagus from similar acid exposure, leaving it intrinsically defenseless comparatively [12,25,27].

Histological and functional mucosal defenses: At the cellular level, the larynx exhibits lower tolerance to acid and pepsin compared to the esophagus, as documented in multiple primary studies. Erickson and Sivasankar used a simulated reflux model to demonstrate that laryngeal mucosa contains thin respiratory epithelium and reduced carbonic anhydrase activity, directly limiting acid neutralization relative to baseline measurements (p<0.05) [22]. In addition, among patients with LPR, decreased E-cadherin expression and dilated paracellular spaces increase epithelial permeability, allowing deeper acid penetration and cumulative injury [22].

In a study conducted by Gill et al., Western blot and immunohistochemical analyses of 54 laryngeal biopsy samples from 18 LPR patients revealed four key findings: (1) intracellular pepsin, a direct marker of refluxate exposure, was found in LPR patients, but undetected in controls; (2) E-cadherin expression was significantly reduced in LPR patients, indicating increased membrane permeability; (3) the larynx’s acid-buffering enzyme, carbonic anhydrase isoenzyme III (CAIII), was absent from the majority of vocal fold epithelium and ventricle samples in LPR patients; and (4) elevated levels of CAIII were found in the epithelium located at the posterior aspect of the commissure, indicating a physiological compensatory response [28]. Together, the accumulation of intracellular pepsin, markedly reduced E-cadherin, and absence of CAIII cause the damage of laryngeal mucosa, paving the way for progressive acid-mediated injury (Figure 3). A 2025 systematic review of human vocal-fold biopsies reported similar findings: cell-junction proteolysis, downregulation of carbonic anhydrase III, and protective mucins (MUC2, MUC4, MUC5AC) were consistent across LPR patients [14]. In addition, Johnston et al. reaffirmed that active pepsin is required for the depletion of squamous epithelial stress protein (Sep70) and CAIII in the laryngeal mucosa [29]. Pepsin was found to linger in the laryngeal epithelium after reflux events, remaining stable until a pH of 8.0, indicating that pepsin can be reactivated due to subsequent decreases in pH, causing a perpetual cycle of laryngeal mucosa injury [29]. By contrast, esophageal mucosa possesses thicker stratified squamous epithelium, submucosal bicarbonate buffering, and robust proliferative repair mechanisms capable of neutralizing acid and pepsin exposure [12,22]. Together, these findings are mechanistically relevant rather than direct comparisons in BN patients. Since primary data are derived from small sample sizes, GERD/LPR patients, or animal experiments, conclusions drawn about disproportional laryngeal vulnerability rest on the convergence of mechanistic evidence rather than a direct BN population mucosal comparison.

**Figure 3:**
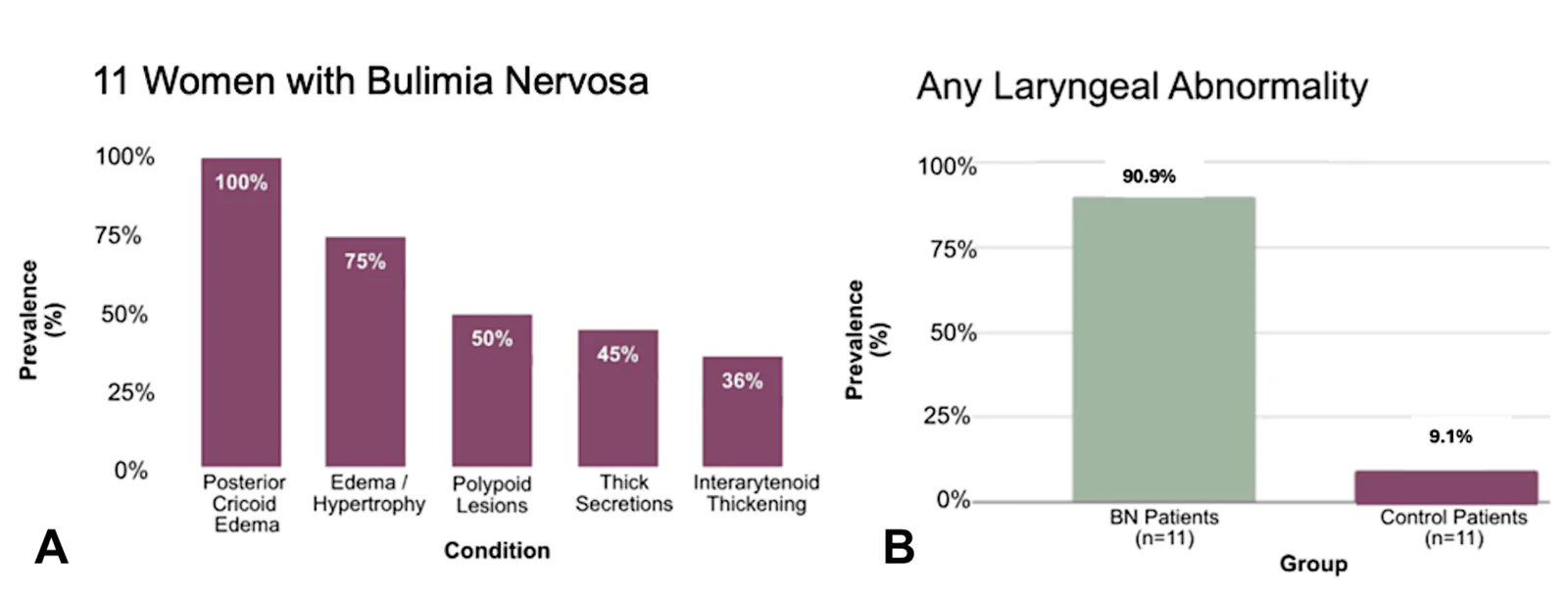
Prevalence of laryngeal findings in bulimia nervosa patients (n=11) vs. controls (n=11) (A) Prevalence of individual laryngeal findings among 11 women diagnosed with BN. (B) Prevalence of any type of laryngeal abnormality in patients with BN versus controls (90.9% vs. 9.1%; χ²=16.010, p=0.001; n=11 per group). Data are extracted from Ferreira et al. [13]. BN: bulimia nervosa

Laryngeal vs. esophageal tolerance thresholds: Two key studies provided direct experimental comparisons between laryngeal and esophageal acid tolerance thresholds. Bulmer et al. used a porcine ex vivo Ussing chamber model to assess laryngeal mucosa integrity. In these mucosa samples, tissue integrity was upheld at pH 4.0, but at pH 2.0, the vocal fold, supraglottic, and subglottic mucosa demonstrated significant compromise at the cellular level (p < .01 and, in some cases, p < .05; CI: 99%), confirming heightened acid susceptibility (Table 2) [21]. Injuries also occurred at higher pH levels when gastric enzymatic factors, such as pepsin, were present. The vocal fold, supraglottic, and subglottic mucosa showed a significant release of cellular components at a pH of 4.0 with pepsin present after just one hour of exposure (P < 0.05), indicating that pepsin, rather than acid alone, is the main factor causing injuries at higher pH levels [21]. Consistent with Gill et al., Bulmer et al.’s experiment identified the subglottic mucosa as the most susceptible site to acid exposure, while the posterior commissure was the least susceptible [21,28]. Bernstein and Baker’s interventional physiological study observed esophageal acid tolerance by infusing hydrochloric acid (pH 1.3) in the esophagus of healthy subjects for about 30 minutes. Results demonstrated that the esophageal mucosa had notable resistance to gastric acidity, with 85% of healthy subjects successfully tolerating a pH of 1.3 [26]. Together, these studies suggest that the laryngeal mucosa is damaged at a greater pH than the esophagus by a difference of about 2.7 pH units. This comparison is limited by differences in species (human vs. porcine), tissue preparation (in vivo vs. ex vivo), and the decade in which the study was conducted.

**Table 2:** Structural and physiologic differences between the larynx and esophagus in acid-mediated injury Visual comparative summary of the anatomical and physiological features that underlie differences in laryngeal and esophageal mucosal vulnerability to acid- and pepsin-mediated injury. Values are drawn from primary and mechanistic literature synthesized in this review (see Table 1 and the Methods section).

Feature	Larynx	Esophagus
Acid Tolerance	Damage at ~pH 2.0; threshold ~4.0 [21]	Tolerates acid to ~pH 1.3 [9,26]
Epithelium Type [12,13,22]	Thin respiratory (2-4 cell layers)	Thick stratified squamous (10-20+ cell layers)
Pepsin-Mediated Mucosal Injury [21,23,29]	Heightened susceptibility to pepsin exposure	Better protection against pepsin injury
Bicarbonate Buffering [12,30]	Limited	Robust
Carbonic Anhydrase Activity [22,28]	Lower	Higher
Healing [12,13,30]	Less robust regenerative capacity	Rapid epithelial turnover and repair

An additional landmark study by Koufman, included as supporting mechanistic evidence, included 225 patients with gastroesophageal reflux disease (GERD) who underwent 24-hour dual-probe pH monitoring. This study revealed that as few as three episodes of refluxate per week produced severe damage to the larynx and that pepsin caused more injury than hydrochloric acid alone [23]. Although Koufman’s findings were derived among patients with GERD rather than BN, the underlying mechanism of injury is directly relevant to BN patients. Whether from reflux or self-induced vomiting, pepsin-mediated laryngeal injury occurs at exposure levels that are too low to damage esophageal mucosa, while the laryngeal mucosa absorbs the brunt of the injury.

When analyzed together in the context of BN, the evidence indicates that repeated exposure of low pH gastric contents may cause greater injury to the laryngopharyngeal mucosa compared with the more resilient esophageal lining [21,23,26]. As such, exposure levels that may be physiologically tolerated by esophageal mucosa are sufficient to induce structural damage in laryngeal tissue [13,30,31]. Moreover, acid exposure alone does not cause laryngeal vulnerability and injury; it is the combination of self-induced vomiting mechanics that drives pepsin and refluxate directly into the larynx, compounding the injury that acid alone would have caused [18,19]. That said, both thresholds indicated were derived from two different study models: Bulmer et al.’s ex vivo porcine tissue versus Bernstein & Baker’s in vivo human infusion; therefore, the two values cannot be directly applied to BN patients. The ~2.7 pH difference should be utilized as an approximation across both studies rather than interpreted as a single measured value.

Laryngopharyngeal Injury

While esophageal injury such as esophagitis is frequently observed in BN-related purging behaviors, laryngeal mucosa demonstrates earlier and more severe reflux-related pathology due to pH sensitivity and reduced buffering capacity as described above [2,32]. Rothstein studied the vocal fold pathology of eight professional singers with BN and confirmed LPR-related findings, including endolaryngeal edema, reflux, posterior commissure hypertrophy, and vocal cord inflammation [24]. Ferreira et al. took these findings one step further. In this prospective case-control study of 22 women (11 with BN and 11 controls; mean illness duration 5.8 years and mean 11.7 purging episodes daily), 100% of BN patients (11/11) exhibited posterior cricoid edema, visually represented in Figure 3 [15]. Patients presented with generalized laryngeal edema and posterior hypertrophy (75%), polypoid lesions or visible capillaries within laryngeal tissue (50%), thick endolaryngeal secretions (45%), and interarytenoid mucosal thickening (36%) (Figure 3) [10,15]. Repetitive acts of purging were also found to have a secretion build-up in 45.5% of patients, likely linked to ciliary dysfunction related to acid erosion and not seen in the control. BN patients were found to have a significantly greater prevalence of laryngeal abnormalities compared to controls (90.9% vs. 9.1%; χ²=16.010, p=0.001; n=11 per group) [15].

In a case-control study by Sunnetci et al., voice changes in 10 BN patients were compared to 10 healthy controls and found that BN patients had significantly elevated voice jitters, shimmers, higher Reflux Finding Scores, and higher Voice Handicap Index-10 scores (p<0.05) [4]. These data provide direct case-control evidence of the measurable impacts that the self-induced vomiting of BN patients has on laryngeal vocal impairment.

Both Ferreira et al. and Sunnetci et al. reported stroboscopic findings of subepithelial hemorrhage, reduced mucosal wave activity, and hyperemia among these patients [4,15]. Across these studies, the larynx was consistently the primary site of acid-mediated mucosal injury in BN due to high rates of chronic irritation resulting from repeated exposure to gastric contents during purging, when compared to the esophagus.

Esophageal Injury

As described in detail above, the esophagus has greater tolerance to gastric contents than the larynx; however, this increased resilience does not prevent the damage induced by long-term purging behaviors. In a literature review, Denholm and Jankowski examined 14 studies that synthesized gastrointestinal complications of BN patients and found that BN patients demonstrated significantly higher rates of reflux symptoms, heartburn, dysphagia, acid regurgitation, and gastrointestinal complaints compared to controls [8]. Several case reports in their review also described the development of Barrett’s esophagus and esophageal adenocarcinoma in long-standing BN patients, which indicates that chronic acid exposure can cause severe esophageal pathology despite its high injury threshold [8]. This is further supported by Gouda and El-Kassas, who described a case study of a 47-year-old male with a 17-year history of binge eating and self-induced vomiting. He presented with severe Grade D GERD (Grade A: mild irritation; Grade D: severe and continuous mucosal breaks with deep ulcers) and an endoscopic biopsy confirmed Barrett’s esophagus with intestinal metaplasia [33]. This diagnosis is the established precursor to esophageal adenocarcinoma. Intestinal metaplasia is considered a higher risk than columnar cell changes without goblet cells. Therefore, if intestinal metaplasia is found, routine endoscopic surveillance is recommended once it is identified [8,33]. Together, these findings demonstrate that while the esophagus requires more frequent and prolonged exposure to gastric contents than the larynx, long-term self-induced vomiting in BN can still break down esophageal mucosa and produce advanced pathology [8,21,26,33]. This clear distinction reinforces the central argument of this systematic review, indicating that both the esophagus and larynx are injured structures in BN patients due to purging behaviors, though the larynx is injured earlier and at much lower acid thresholds than the esophagus.

Limitations

This systematic review has several limitations. First, the studies examining laryngeal pathology among BN patients are limited in number, indicating a broader gap in the number of primary studies conducted for this group. Second, the primary studies utilized had small sample sizes, which limits the generalizability of this review and its findings. Third, narrative reviews were used to provide both general and background information without a risk-of-bias assessment. Fourth, no formal meta-analysis was conducted due to the variation in methodologies, patient populations, and diagnostic tools used in primary studies. Fifth, limiting this review to only English-language literature potentially limited the number of relevant studies included in the screening process. Sixth, Bulmer et al. and Bernstein and Baker had pH threshold comparisons that should be used as supplemental evidence rather than definitive evidence due to the vast methodological differences in the studies. Lastly, this review was not registered on PROSPERO prior to collection of data, limiting publication bias assessments.

## Conclusions

Across all 31 included sources, the larynx demonstrated a consistent pattern of disproportionate mucosal injury relative to the esophagus in BN patients due to self-induced vomiting. This systematic review found three intersecting mechanisms that explain this disparity: (1) anatomical positioning that exposes the larynx to gastric acid without the same defenses the esophagus has; (2) thinner epithelium that is, by nature, more susceptible to damage, with reduced E-cadherin expression, absent CAIII buffering capacity in the presence of refluxate, and intracellular pepsin accumulation; and (3) lower thresholds of acid tolerance, with literature concluding the larynx has damage occurring at a pH of 4.0, while the esophagus can sustain tolerance to the presence of acid at a pH of 1.3 for at least 30 minutes.

Based on observational studies, laryngeal tissue requires significantly fewer acid-exposure episodes to sustain injury as compared with the esophagus. Voice changes, including hoarseness, may precede gastrointestinal symptoms, representing an underrecognized diagnostic window for patients with BN, urging ear, nose, and throat (ENT) protocols for hoarseness and interdisciplinary care to prevent chronic voice dysfunction and progressive throat injury. Most individuals with BN are within normal weight or overweight, frequently resulting in delayed or missed diagnosis, leading to poor prognosis and chronic health problems, further urging early diagnostics and multidisciplinary coordination across gastroenterology, otolaryngology, and mental health disciplines. Further research is warranted to directly compare laryngeal and esophageal mucosa pathology outcomes and pH monitoring in high-risk patients with BN. Prospective studies with longitudinal assessments are needed to evaluate laryngoscopy and pH testing of BN patients before and after receiving treatment to determine long-term outcomes of throat anatomy and pathology once patients have undergone recovery. Moreover, studies replicating the pH findings of Bulmer et al. are necessary to establish acid tolerance thresholds that are clinically applicable. As this patient population is both understudied and often misdiagnosed, interdisciplinary collaboration to enhance diagnostic precision and implement interventional strategies is essential to improve long-term prognoses.
